# Can We Predict Foraging Success in a Marine Predator from Dive Patterns Only? Validation with Prey Capture Attempt Data

**DOI:** 10.1371/journal.pone.0088503

**Published:** 2014-03-06

**Authors:** Morgane Viviant, Pascal Monestiez, Christophe Guinet

**Affiliations:** 1 Centre d'Etudes Biologiques de Chizé, Centre National de la Recherche Scientifique, Villiers en Bois, France; 2 Institut National de la Recherche Agronomique, Unité de Biostatistique et Processus Spatiaux, Domaine St Paul, Site Agroparc, Avignon, France; Texas A&M University-Corpus Christi, United States of America

## Abstract

Predicting how climatic variations will affect marine predator populations relies on our ability to assess foraging success, but evaluating foraging success in a marine predator at sea is particularly difficult. Dive metrics are commonly available for marine mammals, diving birds and some species of fish. Bottom duration or dive duration are usually used as proxies for foraging success. However, few studies have tried to validate these assumptions and identify the set of behavioral variables that best predict foraging success at a given time scale. The objective of this study was to assess if foraging success in Antarctic fur seals could be accurately predicted from dive parameters only, at different temporal scales. For this study, 11 individuals were equipped with either Hall sensors or accelerometers to record dive profiles and detect mouth-opening events, which were considered prey capture attempts. The number of prey capture attempts was best predicted by descent and ascent rates at the dive scale; bottom duration and descent rates at 30-min, 1-h, and 2-h scales; and ascent rates and maximum dive depths at the all-night scale. Model performances increased with temporal scales, but rank and sign of the factors varied according to the time scale considered, suggesting that behavioral adjustment in response to prey distribution could occur at certain scales only. The models predicted the foraging intensity of new individuals with good accuracy despite high inter-individual differences. Dive metrics that predict foraging success depend on the species and the scale considered, as verified by the literature and this study. The methodology used in our study is easy to implement, enables an assessment of model performance, and could be applied to any other marine predator.

## Introduction

Predicting the effect of oceanographic and climatic variation on top marine predator populations and/or establishing management decisions (i.e., marine protected areas) are often based on resource selection analyses and habitat modeling output [1], [2], [3], [4], [5]. Understanding where, when, and how animals forage is essential to identify their habitat and assess the likely effect of climatic or anthropogenic changes on both reproductive success and/or survival. A growing number of studies are focused on characterizing foraging habitats for numerous marine species [2], [3], [4], [6]. However, this task is particularly challenging in the marine environment because of the high spatial and temporal dynamics of oceanic systems and the difficulty of observing these predators at sea.

Tracking information for animals at sea is provided by a broad range of telemetric tools such as Argos satellite system, Global Positioning System, or light-based geolocation sensors. Residence time (estimated as time spent per unit area), first passage time, or state–space modeling approaches are commonly used to infer foraging behavior from tracking data [6], [7], [8], [9], [10]. These indices are based on the assumption that animals will increase the time spent searching for food in more profitable prey patches [11], thus reducing their speed and increasing their turning angles [12]. However, depending on the species and environmental conditions, inferring foraging success from tracking data is not always possible [13], [14], [15] and could result in misleading assessments of habitat selection [16].

For diving predators, several studies have used dive metrics such as dive duration, bottom duration, or a combination of dive metrics to infer foraging activity and prey patch quality visited [17], [18], [19] assuming that the predator forages longer at the bottom of dives in a better patch quality [20]. Based on this assumption, several studies used dive classification to assign different dive shapes to different behaviors: foraging, transiting, and resting [21]. The amount of time spent at the bottom of a dive for a given location was found to correlate well with increasing body condition in southern elephant seals, *Mirounga leonina*, assessed by monitoring drift dives [22].

However, these studies lack concomitant information on prey ingestion rate. In recent years, several studies have tried to address this deficiency by using a range of new loggers to assess prey ingestion. Esophageal and stomach temperature sensors [23], [24], Hall sensors, or accelerometers to detect mouth opening events [25], [26], [27] as well as video cameras [27], [28], [29] have been used to investigate fine-scale foraging success in marine predators.

Unfortunately, these loggers can be difficult to deploy, or because of their size (such as the Crittercam [28]), they may negatively affect the foraging efficiency of these predators. Furthermore, they collect vast amounts of data that require long and tedious analyses. For these reasons, loggers are generally deployed for a limited number of individuals in the field, making inference at the population level difficult. Therefore, the development of reliable and simpler behavioral indices of foraging success based on diving patterns alone would not only enable working with larger sample sizes but also revisiting the long-term time series of diving behavior that is available for some species to initiate retrospective studies.

Some attempts to identify such behavioral indices have been made. Several studies have tried to relate foraging success (or effort) or quality of the prey field encountered to the diving patterns of individuals. The bottom phase of dives has been validated as the time when most feeding occurs in several species: Antarctic fur seals (*Arctocephalus gazella*) [29], northern elephant seals (*Mirounga angustirostris*) [30], grey seals (*Halichoerus grypus*) [31], Magellanic penguins (*Spheniscus magellanicus*) [24], Weddell seals (*Leptonychotes weddellii*) [32], and leatherback turtles (*Dermochelys coriacea*) [33]. Mori et al. [34], [35] showed that the proportion of residence time (bottom time) in relation to standard time (optimal time spent at the bottom of a dive for a given depth) correlates well with prey patch richness (estimated using cameras) in Weddell seals. In grey seals, accumulated bottom duration best predicted the number of feeding events at the dive bout scale [36]. Other studies show that feeding events for different penguin and whales species are well correlated to number of wiggles in the dives [37], [38], [39], [40], [41], [42].

The objective of this study was to build predictive models of foraging success in Antarctic fur seals (through information on prey capture attempts detected by accelerometers or Hall sensors) using diving data information only. The goal and originality of the study are based on the following approach: testing a more complete set of behavioral parameters (including dive metrics such as transit and resting times), not just those initially suspected to be linked to foraging success (bottom duration and wiggles); evaluating the effect of the time scale considered; and, more importantly, assessing the predictive power and accuracy of these models and their applicability to new, unknown individuals.

## Materials and Methods

### Ethic statement

Our study on Antarctic fur seals was approved and authorized by the ethics committee of the Centre National de la Recherche Scientifique (CNRS) and the French Polar Institute (Institut Paul Emile Victor – IPEV- Comité de l'Environnement Polaire) in May 2007. These Institutes do not provide any permit number or approval ID, however animals were handled and cared for in accordance with the guidelines and recommendations of these committees (dirpol@ipev.fr). Manipulations of animals were conducted under the “authorization of experimentation for vertebrate species” of Christophe Guinet (n°7200). Handling time for equipment deployment was less than 20 minutes, and less than 10 minutes for equipment recovery.

### Study Site

Data on diving and foraging behavior in Antarctic fur seals were collected at Pointe Suzanne (49°S, 70°E) in the Kerguelen Islands during the breeding seasons (December to February) of 2007–2008 and 2008–2009. Antarctic fur seals tend to perform shallow and short dives compared to most diving predators but the population present in Kerguelen Island dive deeper than other population of the same species [43]. In the Kerguelen Islands, Antarctic fur seals feed mainly on small myctophid fish (5–10 cm), with *Gymnoscopelus* spp. and *Electrona subaspera* representing 60% and 20%, respectively, of the diet [44], [45]. Myctophids perform day–night migrations and are accessible to diving fur seals only at night, when they are close to the surface [46].

### Instrument deployment

Accelerometers (M190L-D2GT, Little Leonardo, Tokyo, Japan, 60 mm in length and 15 mm in diameter, 18 g in air, i.e. less than 0.1% of animal weight) and intermandibular extension sensors (Hall sensors; developed by the Centre d'Ecologie et Physiologie Energétique, Centre National de la Recherche Scientifique, Strasbourg, France, 80×20×12 mm, 55 g in air, less than 0.2% of animal weight) were deployed on adult lactating females to study their diving activity and to detect mouth-opening events. The Hall sensor logger operated by attaching a magnet to the animal's upper jaw and a Hall sensor to the opposite mandible and recording variation in the electromagnetic field induced by mouth openings [25]. The accelerometer logger, attached to the fur under the animal's lower jaw, detects horizontal and vertical accelerations and recorded the sudden acceleration changes induced by mouth openings [26], [27]. Hall sensor units and accelerometers were set to sample at frequencies of 16 Hz. Depth sensors (±0.1 m) were integrated into both loggers and sampled at 1 Hz.

Lactating females were captured onshore during their nursing visits (using a hoop net), weighed (±0.2 kg), and measured (straight-line length, ±0.5 cm). While the animals were under gas anesthesia (using isoflurane) following Gales and Mattlin procedure [47], the sensors were glued to their heads. Nine females were equipped with accelerometers, and 4 females were equipped with Hall sensor units. The devices were mounted on nylon webbing with cable ties and were glued to the fur with a two-part, fast-setting araldite (Araldite AW 2101, Ciba). The Hall sensor was placed on the animal's lower jaw, and the magnet was glued to the upper jaw, just in front of the sensor. A cable was used to connect the Hall sensor to the main recording unit glued to the top of the head. The devices were recovered by cutting the fur just under the glued loggers after a single foraging trip.

### Dive analyses

The time series of diving behavior was reconstructed using a custom-developed R program [48]. Depth readings were corrected from the pressure transducer surface offset. As offsets were found to vary over a given foraging trip a fitted trend was applied to all surface records. A surface record was defined as the minimum pressure values between two dives. Individual dives were defined as any depth exceeding 3 m from surface [43]. However, previous studies described a bimodal distribution of dive depths for Antarctic fur seals in the Kerguelen Islands separated by a 15-m depth threshold [43]. Most nights, fur seals dove deeper than 15 m. Furthermore, accelerometer data analyses revealed that accelerometer signal intensity and duration were different above and below 15 m, suggesting that fur seals were targeting different prey items during shallower dives (unpublished data). Precise diving parameters could not be extracted from dives shallower than 15 m because dive duration in such shallow depths is short. Thus, only dives deeper than 15 m were considered for establishing predictive models. For those dives (deeper than 15 m), recorded parameters included time at the beginning and end of the dive, maximum depth (m), descent duration (s), bottom duration (s), ascent duration (s), dive duration (s), and post-dive surface interval (s). Termination of descent was defined as the point at which the rate of a continuous descent was less than 0.4 m/s. Ascent start was defined as the point at which the rate of a continuous ascent exceeded 0.4 m/s. This value corresponded to a threshold after which a net change in descent and ascent rates was observed and was empirically validated on the entire data set. We defined duration as the difference between the end of the descent and the beginning of the ascent. Descent rate was defined as depth at the start of the bottom phase divided by descent duration, and ascent rate was defined as depth at the end of the bottom phase divided by ascent duration.

**Figure 1:**
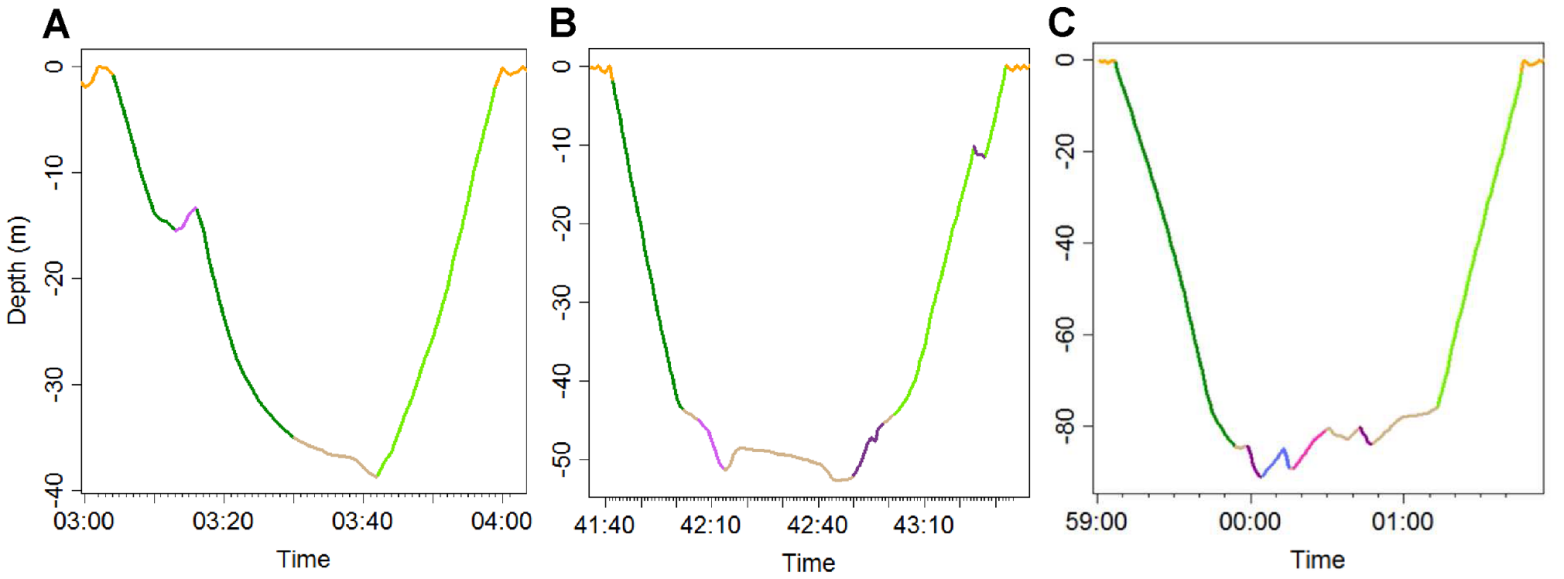
Dive profiles. Descent, bottom, and ascent phases are in dark green, cream, and light green, respectively. Steps in the descent phase and descending steps in the bottom phase are in light purple. Steps in the ascent and ascending steps in the bottom phase are in dark purple. Wiggles are in blue.

**Figure 2:**
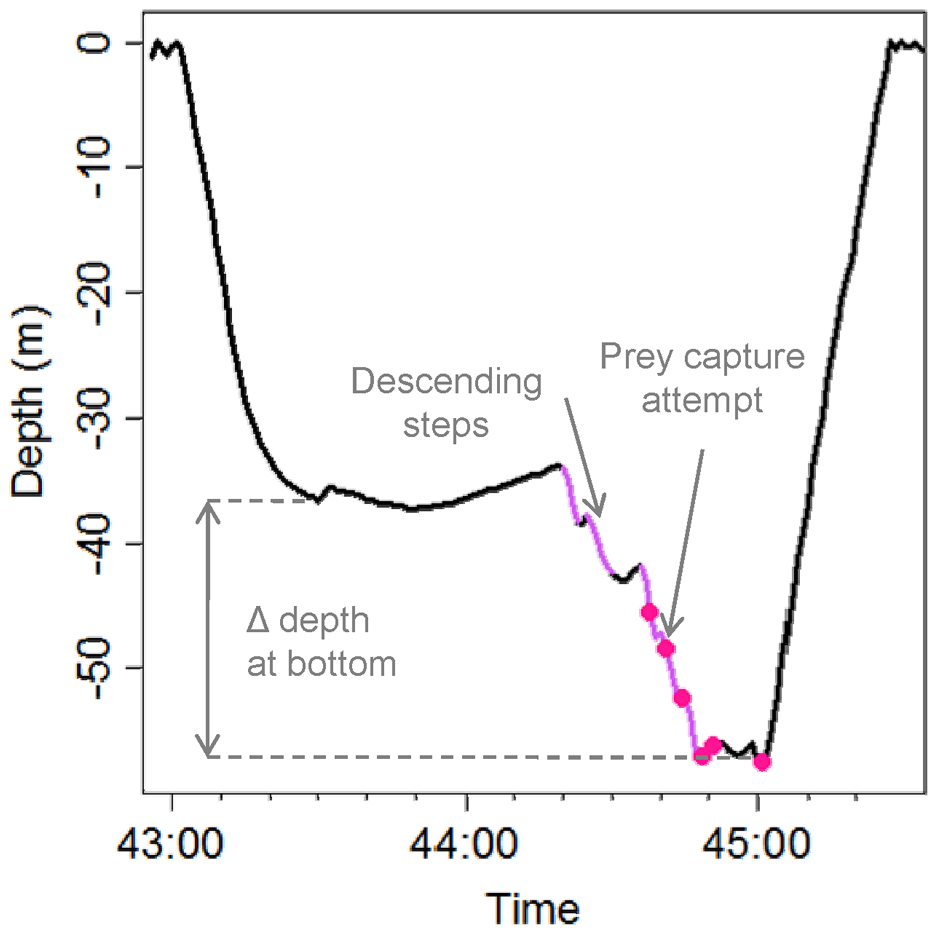
Dive profile with several steps descending in the bottom phase (light purple). These successive steps generally lead to a high variation of depth in the bottom phase. Prey capture attempts are symbolized by pink dots.

Steps in the descent and ascent were defined as the instantaneous rate of change of depth less than 0.4 m/s for less than 8 s and occurring before 60% of the maximum dive depth (Figure 1A, B) as steps occurring at a greater percentage of diving depth resulted in the initiation of the bottom phase of the dive (i.e. mean vertical descent speed lower than 0.4 m.s^−1^). Descending steps in the bottom phase were characterized by negative changes of depth greater than 0.4 m/s and lasting less than 8 s, and ascending steps were characterized by positive changes of depths greater than 0.4 m/s and lasting less than 8 s (Figure 1C). Wiggles were defined as the succession of an ascending and descending step in the bottom phase separated by less than 3 s (Figure 1C). An index of variation of depth at the bottom of dives was calculated as the difference between maximum dive depth and depth at the start of the bottom phase (Figure 2).

Sequential dives were allocated into dive bouts according to Luque and Guinet [49], in which the bout-ending criterion is determined using maximum likelihood estimation and is based on the absolute difference of post-dive interval duration. Only dives occurring within bouts of at least 3 dives were included in the analyses, which excluded solitary isolated dives. We also excluded isolated dives at the end of a diving bout (i.e., those not followed by another dive), since post-dive intervals probably correspond to behaviours other than post-dive recovery.

### Determination of mouth-opening events

Because definitively linking mouth openings to true prey ingestion was not possible, we considered mouth-opening events to reflect prey capture attempts. Changes in the number of prey capture attempts between dives are thought to reflect intensity of prey encounters. Mouth-opening events were detected using acceleration data from the lower jaw following the method described by Viviant et al. [25]. Horizontal accelerations recorded on the animal's lower jaw were first filtered with a 3-Hz high-pass filter to smooth out low-frequency acceleration of the head and body. This highlighted the peaks in accelerations due to mouth openings. Variances were calculated for a moving window of 1.5 s and highlighted extreme accelerations considered to be true mouth-opening events. A similar analysis was performed on the Hall sensor data using a moving window of 5 s (a wider window was necessary to accommodate the time required for the sensor to return to its basal value after a mouth-opening event). The number and time of occurrences of mouth-opening events were routinely identified for each dive.

### Model structure and selection of predictors

We used a hierarchical modeling approach to identify diving parameters that most accurately reflected the foraging state of the individuals. Generalized linear mixed models (GLMM) were constructed using the “lmer” function in the R package [48] to relate the number of prey capture attempts (response variable) to diving characteristics (explanatory variables). Maximum dive depth, surface duration, descent and ascent rates, bottom duration, variation of depths at the bottom, steps in the descent and ascent phases, steps descending in the bottom phase, steps ascending in the bottom phase and wiggles in the bottom phase were selected as explanatory variables.

Prior to modeling, all diving variables were standardized (centered and scaled) to facilitate model convergence and comparison of the predictor scales [50]. Models for prey capture attempts which correspond to discrete events (counts) were fitted with a Poisson distribution, and a log link function. Individual identity was included as the random intercept term to account for the hierarchical structure of the data.

### Model selection and inference

We performed all possible linear combinations of explanatory variables and ranked the models using Akaike's Information Criterion (AIC) [51]. The Akaike weight of each model was then calculated as a representation of the relative likelihood of candidate models [52]. The candidate models selected were those for which the sum of the Akaike weights was greater than 0.95. When the analyses indicated more than 1 candidate model, a model averaging procedure was conducted based on the parameters of the candidate models and their weights [52].

### Prediction scales

Models were run first at the dive scale and then at the dive bout and all-night scales. For these larger scales, averaged diving parameters were used as predictors, and number of dives was added as an additional explanatory variable. Because dive bout duration and number of dives were highly variable among bouts, predictive models were also established for fixed time scales of 30-min, 1-h, and 2-h intervals inside dive bouts.

At the night scale, only complete nights (i.e., nights during which the logger did not stop recording) were selected. Because of differences in foraging behavior between dives shallower and deeper than 15 m, at the dive bout scale and at the 30-min, 1-h, and 2-h sequences within a bout, analyses were conducted only on sequences in which the cumulated dives cycle duration of dives greater than 15 m represented more than 85% of the entire sequence duration. This percentage was added as an explanatory variable in the models.

Generalized linear mixed models (GLMMs) for prey capture attempts fitted with a Poisson distribution were used for all time scales except the night scale. A GLM with a Poisson distribution was used for the night scale because the small sample size prevented use of a random effect on the individuals. To avoid overfitting the model during the selection procedure, the maximum number of explanatory variables that could be tested at one time was dependent on the sample size of each time scale: 11 (all) variables at the dive scale, 7 variables at the dive bout scale, 13 (all) variables at the 30-min and 1-h scales, 4 variables at the 2-h scale, and 2 variables at the night scale. This design permitted a minimum of 10 data points per explanatory variable tested. At the dive bout, night, 30-min, 1-h, and 2-h scales, AICc was used to avoid overfitting and to account for small sample size [51]. At the dive scale, AIC and AICc were strictly equivalent because of the large sample size, so AICc was used and presented for consistency.

Residuals and relationships with fitted values were checked, but no distinct patterns were observed (results not presented). Generalized additive mixed models (GAMMs) were also tested to account for the nonlinearity of 2 variables but did not significantly improve foraging success predictions. Because the effort of model fitting and computational costs are much higher for a GAMM compared to a GLMM and both models produced similar predictions, the GLMMs were preferred.

### Model evaluation

We conducted cross-validations to assess predictive performances (model with lowest AIC or model averaging). A “leave-one-out” procedure was chosen; this procedure consisted of fitting the models on all individuals minus one and then applying the predictors to the remaining individual for predictions. All possible combinations of individuals for cross-validation were tested.

The predictive performances of models were assessed by comparing observed and predicted number of prey capture attempts using the concordance index (C-index; Hmisc package) [53]. The C-index estimates the probability of concordance between predicted and observed responses. The C-index varies from 0.5 to 1, with the following model predictive performance classification: greater than 0.9, excellent; 0.9–0.8, good; 0.8–0.7, reasonable; 0.7–0.6, poor; and 0.6–0.5, unsuccessful [53], [54]. Results are presented as mean ± standard deviation, unless stated otherwise.

## Results

### General diving and foraging characteristics

Foraging trip of the 11 Antarctic Fur seal females lasted 9.5±3.1 days (n = 11). A total of 5384 dives from the 11 individuals were analyzed: 2106 dives from individuals equipped with accelerometers and 3278 dives from individuals equipped with Hall sensors (More dives were recorded for animals equipped with Hall sensors compared to Accelerometers due to their larger memory size). A total of 3056 dives were associated with at least 1 prey capture attempt (57%) while the mean number of prey capture attempts per dive was 1.2±1.8 (range: 0–15).

Descriptive statistics on the numbers of diving bouts or dive sequences (with more than 85% of dives greater than 15 m), dives and prey capture attempts are summarized in Table 1.

**Table 1:** Sample sizes, number of dives and number of prey capture attempts measured at different temporal scales.

	Number of bouts or sequences	Number of dives ± SD (range)	Number of prey capture attempts ± SD (range)
Bout	72	52±49 (4–181)	67±80 (24–215)
30 min seq.	239	13±4 (5–31)	18±11 (0–68)
60 min seq.	115	72±29 (19–162)	36±18 (5–105)
120 min seq.	39	54±13 (36–94)	72±29 (19–162)
Night seq.	16	151±41 (93–240)	183±68 (59–302)

### Best diving predictors of foraging activity

**Figure 3:**
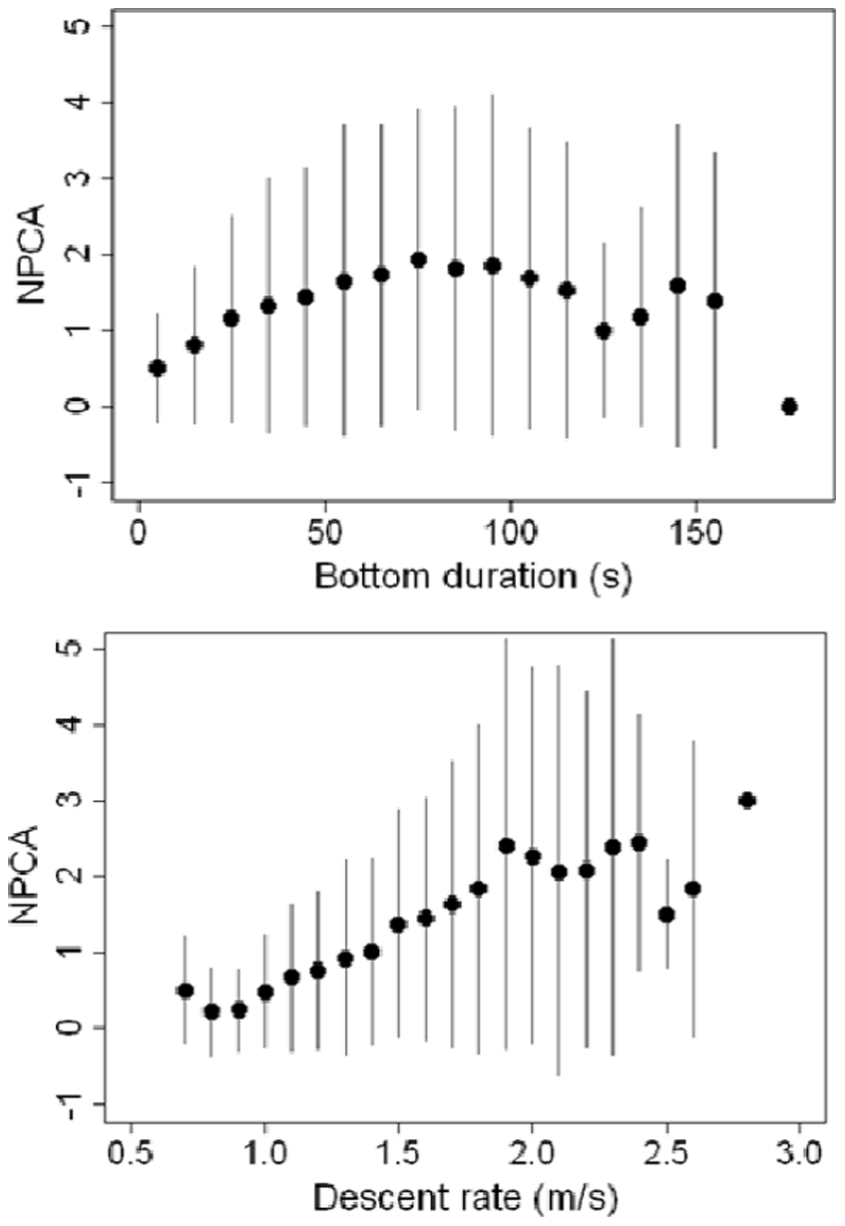
Effect of bottom durations (top) and descent rates (bottom) on number of prey capture attempts. The number of prey capture attempts per dive was used.

The ranking of alternative models at the dive scale (Model details Table S1A) showed that the number of prey capture attempts was significantly and positively affected by descent rate, ascent rate, number of descending steps at the bottom of the dive, surface duration, maximum dive depth, number of steps during descent and ascent, depth variation at the bottom of the dive, and bottom duration (to a lesser extent) (Figure 3; Table 2). Descent and ascent rates were the most important predictors, and bottom duration had only a weak positive influence (Table 2).

**Table 2:** Results of model averaging based on the best predictive models for number of prey capture attempts at different temporal scales.

	Dive		Bout		30 min in Bout		1 Hour in Bout		2 Hours in Bout		Night	
Final Models	w	Coef ± SE	w	Coef ± SE	w	Coef ± SE	w	Coef ± SE	w	Coef ± SE	w	Coef ± SE
Intercept	1	0.058±0.073	1	3.57±0.14	1	2.76±0.07	1	3.42±0.09	1	4.18±0.08	1	5.16±0.02
Descent rate	1	0.240±0.020	1	0.46±0.04	1	0.35±0.03	1	0.36±0.04	1	0.32±0.06	0	0
Bottom duration	0.70	0.025±0.015	0.94	−0.18±0.05	1	−0.28±0.05	1	−0.40±0.06	1	−0.30±0.08	0	0
Ascent rate	1	0.194±0.016	1	0.24±0.05	1	0.13±0.03	0.9	0.10±0.04	0	0	1	0.23±0.02
Maximum dive depth	1	0.091±0.021	0	0	1	−0.20±0.05	0.97	−0.19±0.06	0.03	0.004±0.004	1	0.19±0.02
Surface duration	1	0.162±0.021	0	0	1	0.18±0.05	0.86	0.12±0.05	0.08	0.008±0.008	0	0
Steps descending in the bottom	1	0.143±0.015	1	0.39±0.04	1	0.08±0.02	0.27	0.004±0.01	0.46	0.06±0.03	0	0
Steps ascending in the bottom	0.35	0.004±0.006	1	−0.23±0.05	0.63	0.03±0.02	0.38	0.02±0.02	0.02	0.002±0.002	0	0
Steps in the ascent	1	0.065±0.009	1	0.12±0.03	1	0.06±0.02	0.98	0.05±0.02	0	0	0	0
Steps in the descent	1	0.075±0.010	0	0	0.68	−0.02±0.01	0.22	0.002±0.005	0	0	0	0
Depth variation at bottom	1	0.074±0.015	0.05	−0.01±0.01	0.26	0.002±0.007	0.73	0.04±0.02	0	0	0	0
Wiggles in the bottom	0.35	0.004±0.005	0	0	0.29	0.004±0.007	0.43	0.02±0.01	0.02	0.002±0.002	0	0
Nb of dives	NI	NI	1	0.71±0.02	0.97	−0.15±0.05	1	−0.25±0.06	0.43	−0.091±0.056	0	0
% of time for dives >15 m	NI	NI	0	0	1	0.10±0.02	1	0.18±0.02	0.96	0.12±0.03	0	0
**C-index**												
C-index for Complete data set		0.70		0.84		0.77		0.79		0.81		0.88
C-index for Cross-Validation		0.69		0.82		0.73		0.74		0.76		0.72

Coefficient of predictive models developed at each time scale (mean ± standard error). All models used for model averaging are generalized linear mixed models except at the night scale where generalized linear models were used. The concordance index (C-index) is shown for final averaged models on complete data sets and for cross-validations. NI: nonincluded predictor; NS: nonselected predictors. Explanatory variables correspond to raw values for the dive scale and to mean values for greater scales, except the number of dives and percent of time (cumulated dive cycle duration) for dives greater than 15 m.

At the dive bout scale, the number of dives and mean descent rates were the most important predictors of the number of prey encounters per bout (Table 2; Model details Table S1B). Ascent rate, steps descending and ascending in the bottom phase, bottom duration, and steps in the ascent were less important predictors. Surprisingly, bottom duration had a negative influence on the number of prey capture attempts (Table 2).

For all fixed time scales in the dive bouts (30 min, 1 h, and 2 h), the ranking of alternative models (Model details Table S1C,D,E) showed that the best predictors of the number of prey capture attempts were descent rate and bottom duration (Table 2). At all scales, bottom duration had a negative effect on the number of prey capture attempts. At the 30-min and 1-h scales, maximum dive depth and number of dives were also important negative predictors, and surface duration, percentage of time (dives cycle duration) for dives greater than 15 m, ascent rate, and steps in the ascent were important positive predictors (Table 2).

The two most important predictors of the number of prey capture attempts per night were ascent rate and maximum dive depth, which had a positive influence (Table 2; Model details Table S1F).

### Performance and individual variability of models

**Figure 4:**
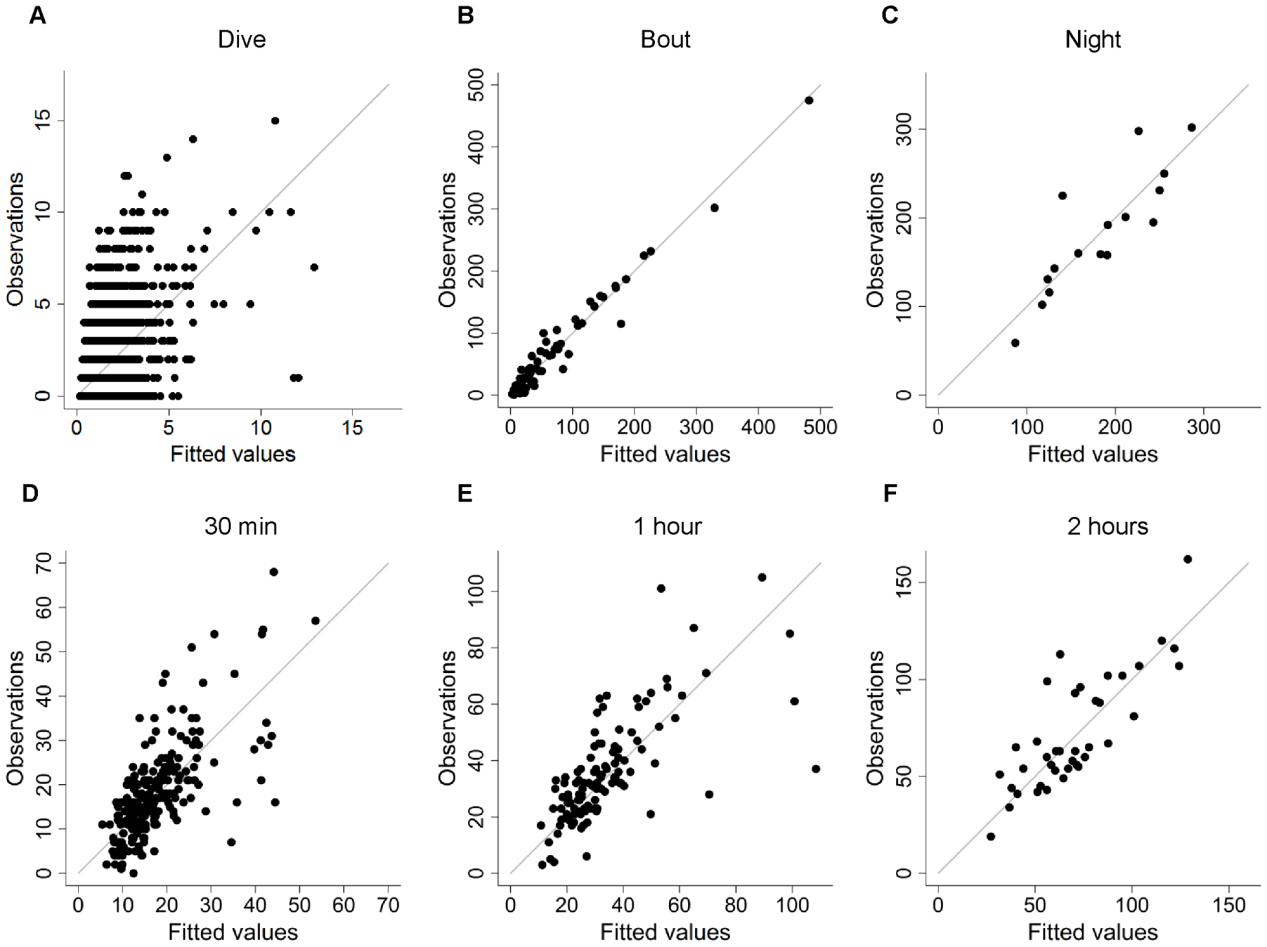
Observations versus fitted values of prey capture attempts on the entire data set. This relationship is shown at the dive (A), dive bout (B), night (C), 30 min (D), 1 h (E) and 2 h (F) scales. Final averaged generalized linear mixed models were used to estimate fitted values at every time scale with the exception of the night scale, where a generalized linear model was used.

**Figure 5:**
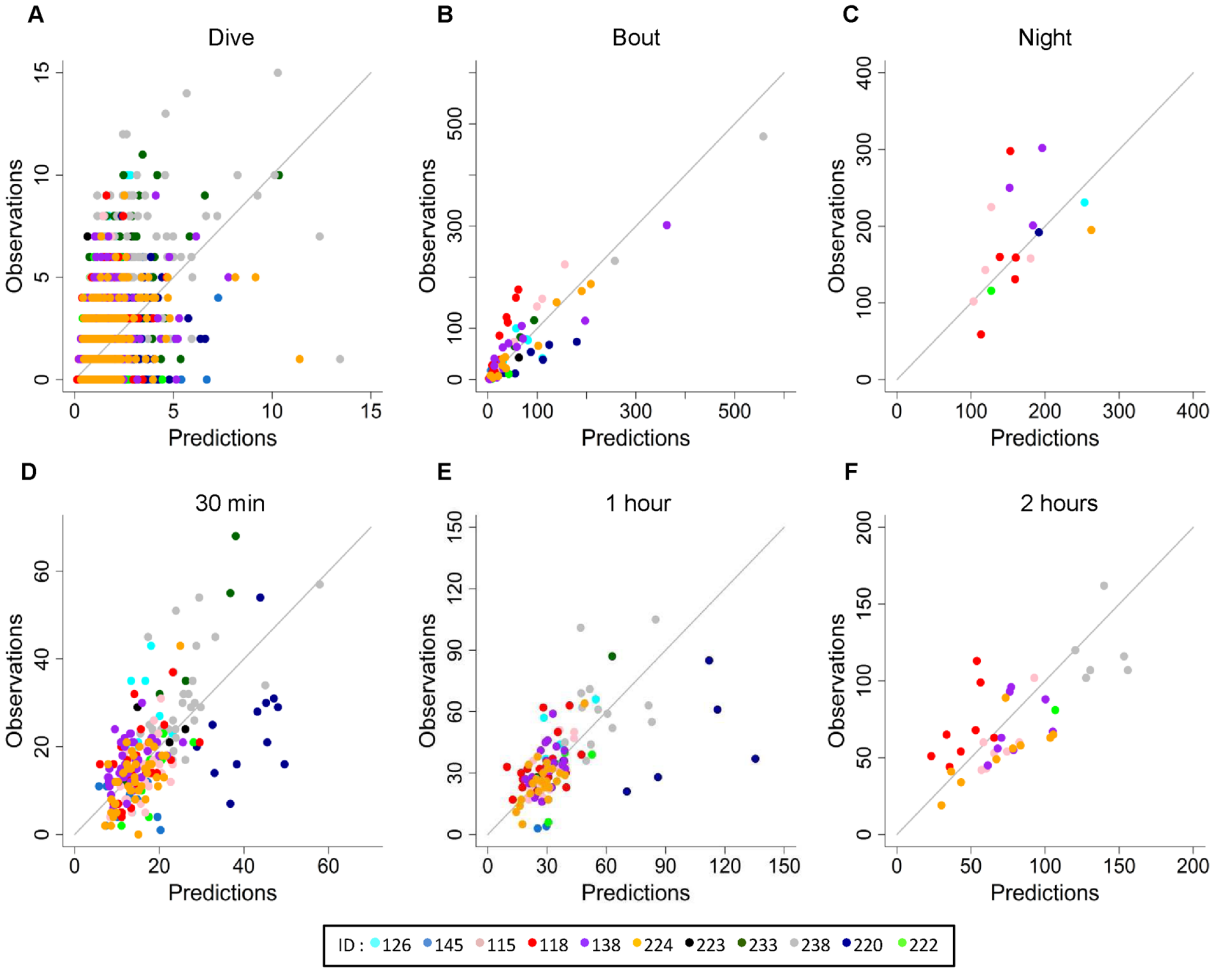
Observations versus predicted values of prey capture attempts for “leave-one-out” cross-validations. These relationships are presented for the dive (A), dive bout (B), night (C), 30 min (D), 1 h (E) and 2 h (F) scales. Generalized linear mixed models were used for all time scales except the night scale, where a generalized linear model was used. Each color represents a different individual (ID).

The model at the dive scale showed poor model performance (C-index = 0.70) for the complete data set (Figure 4A) as well as for cross-validations on the test data set (C-index = 0.69; Table 2; Figure 5A).

At the dive bout scale, the models were able to explain an important part of variation in foraging intensity (C-index = 0.84) for the complete data set (Figure 4B). Performance decreased for cross-validations on the test data set but still performed well (C-index = 0.82; Table 2; Figure 5B).

Cross-validation output indicated that foraging patterns were best predicted for increasing time scales (Table 2). At the fixed time scales of 30 min, 1 h, and 2 h, all models showed reasonable to good predictive performances for the complete data set (C-index = 0.77, 0.79, and 0.81, respectively) as well as for the cross-validation on the test data set (C-index = 0.73, 0.74, 0.76, respectively; Table 2; Figures 4D,E,F and 5D,E,F). The model at the night scale also exhibited very good predictive performance (C-index = 0.88; Figure 4C) on the complete data set and reasonable performance during cross-validations (C-index = 0.72; Table 2; Figure 5C).

### Individual variability

The C-index values of the cross-validation indicated that, globally, foraging models established on training individuals could predict the foraging patterns of test individuals. However, the prediction for 1 individual (ID = 220) was overestimated (Figure 5D,E; in dark blue).

## Discussion

We assumed in this study that the number of prey capture attempts was representative of foraging success (assuming that missed attempts occur at a constant proportion relative to successful attempts). We thus refer to foraging success later in the discussion. Our study shows that several diving parameters can be used at different temporal scales to predict with reasonable accuracy foraging success in Antarctic fur seal females breeding on Kerguelen Islands. Yet, the order of importance of diving predictors changes depending on the time scale investigated.

The effect of bottom duration on foraging success was expected as many studies have found that foraging takes place during the bottom phase of dives. At the dive scale however, bottom duration was a poor predictor of foraging success. In accordance with the Charnov theorem [55], the number of prey capture attempts was found to be an increasing but decelerating function of bottom duration in fur seals (Figure 3). However, we unexpectedly found the opposite relationship at greater temporal scales, with the number of prey capture attempts decreasing with increasing bottom duration. Furthermore, bottom duration became one of the highest predictors of foraging success at greater time scales (30 min, 1 h, and 2 h). This finding suggests that Antarctic fur seals adapt their diving behavior to the prey field encountered and tend to reduce time spent at the bottom of dives for long and successful diving bouts. The finding that Antarctic fur seals tend to decrease bottom duration when successful at increasing time scales tends to suggest that successful foraging dives are energetically more costly than unsuccessful ones, as a consequence Antarctic fur seals must return to the surface earlier compared to the longer bottom duration of unsuccessful dives. This action likely represents a behavioral response to maintain aerobic metabolism and to reduce the risk of accumulating a large oxygen debt with increasing duration of foraging bouts. This behavior should be investigated further by monitoring swimming effort using accelerometers and heart rate between successful and unsuccessful dives.

Although wiggles correlated well with foraging activity for several penguin and whale species [37], [38], [39], [40], [41], [42], in our study, this parameter was unrelated to foraging success in Antarctic fur seals at any time scale. However, the number of descending steps during the bottom phase of a dive was a good predictor of the number of prey capture attempts in fur seals at different scales (dive, 30 min, and 2 h). This result suggests that fur seals were probably chasing their prey from above (Figure 2). Moreover, descending steps were often observed in succession, suggesting that Antarctic fur seals were pursuing their prey, which were probably moving to deeper waters to escape (Figure 2). This observation indicates that the decelerating function of the number of prey capture attempts with increasing bottom duration at the dive scale could be related to prey escape and/or dispersion behavior rather than prey patch depletion.

Steps occurring in the descent and ascent phases were also associated with increasing foraging success. However, these events represented less than 20% of all prey capture attempts (unpublished data), which explains the low predictive power of these variables.

Some marine predator species have been shown to adjust their behavior during the transit or resting phase of a dive cycle according to their recent foraging success or prey encounters [56], [57]. Despite these observations, transit rates (descent and ascent) and recovery time (surface duration) were overlooked in studies trying to predict foraging success in marine predators by considering the diving portion of a dive cycle (dive+surface interval). To our knowledge, we show for the first time that vertical transit rates and recovery time at the surface are important predictors of foraging activity in a marine predator.

Unexpectedly, descent rate was found to be the most constant and reliable predictor of foraging success regardless of the time scale considered. Descent rate was the first or second highest predictor of the number of prey capture attempts at every time scale with the exception of the night scale, where ascent rate was the best predictor. This finding suggests that Antarctic fur seals may continuously adjust their transit rates according to previous foraging success, and they may anticipate future foraging success by increasing descent rate to return rapidly to the prey patch. Indeed, as fur seal swims faster to the surface consecutively to successful bottom phase of the dive and then descend quicker during the following dive, suggests that they indeed adjust their descent behavior in relation to the foraging success of the previous dive. Alternatively, fur seal might use other sensory systems such as acoustic or visual signals, such as bioluminescence, produced by their prey to assess the quality of prey patch from the surface, but such hypothesis remains to be tested. The quicker descent rate is likely related to a steeper descent angle rather than increasing swimming speed to return to the same prey patch location, as was found for Adélie penguins (*Pygoscelys adeliae*) [58] and Little penguins (*Aptenodytes patagonicus*) [57]. Future work will investigate that issue. Interestingly, increasing surface duration was also found to relate positively to the number of prey capture attempts. This relationship suggests that prey chase is costly and may require longer time recovering at the surface or in anticipation of a longer foraging dive.

### Diving intensity

Taking into account the maximum diving depth, the number of dives within a given time scale was found to be negatively related to foraging success, suggesting that increasing diving activity per unit time is indicative of lower foraging success and/or foraging in low-quality patches. This result is in agreement with the adjustment of bottom duration we found at these time scales: In poor quality patches, Antarctic fur seals increase their foraging effort by increasing their diving intensity and the time spent searching for prey at the bottom of dive.

### Effect of scales on model performance

The predictive abilities of our model were found to increase according to the time scale considered. Model performance was relatively low at the dive scale but became reasonably good at time scales of 30 min and greater. These changes in model performance and in the importance of predictor variables according to each time scale suggests that some behavioral adjustments are more predominant at some scales than at others or that Antarctic fur seals may be sensitive to the distribution or availability of some prey at certain scales only. A change in diving predictors according to time scales was also observed in grey seals [36].

### Individual variability and population inference

When trying to understand which diving patterns are associated with feeding activity in a marine predator species, the common assumption is that patterns for an individual can be generalized to the whole population. However, because the number of individuals available in tracking studies generally is small compared to population size, accounting for interindividual variability is important. Our modeling approach enabled us to assess the effect of individual variability when predicting foraging activity in new individuals. Our cross-validation exercise confirmed the relatively high inter-individual differences. Among those differences, 1 individual (ID 220) showed particularly high mean descent rates, which resulted in an overestimation of its prey capture attempts. However, ten of the eleven individuals showed good predictions at scales equal to or greater than 30 min, indicating that models were globally capable of accurately predicting foraging activity in new individuals based on diving patterns alone. This finding is important and suggests that we should be able to estimate inter-individual as well as inter-annual differences in foraging success in breeding Antarctic fur seal females foraging in the Kerguelen Islands from dive patterns only. Therefore, monitoring changes in foraging behavior could be a useful bio-indicator of environmental quality and environmental risks caused by climatic changes. Behavioral indicators can be used to detect changes more quickly than a demographic survey because environment will affect behavior well before a change in demographic trait is detected [1]. We would expect inter-annual changes in foraging success to relate well and rapidly with pup growth differences. Behavioral indicators established in this study could constitute a useful, simple ecological tool for population monitoring or conservation purposes [1]. This study opens new perspectives for investigating the consequences of inter-annual variations in environmental conditions on foraging efficiency in Antarctic fur seals. Indeed large diving data sets, collected at Kerguelen Islands since 1998 and under contrasted oceanographic conditions are already available to conduct such analysis.

Importantly, to predict the foraging success of Antarctic fur seals, if we had assumed that their foraging success was positively related to the bottom duration or the number of wiggles, because these relationship were found in other marine predator species [36], [39], [42], this would have resulted in very poor predictions at the dive scale and false predictions at greater temporal scales !

Thus, before using some diving patterns, the validity of the predictors for the species considered must be assessed. Our study also revealed that vertical transit rates and recovery time at the surface were important predictors of foraging success in a marine predator. We believe that behavioral adjustments observed in Antarctic fur seals may appear in other predators and that the importance of these parameters should be investigated in other species.

## Supporting Information

Table S1 **Results of model selection at every time scales.** Models are generalized linear mixed models at all time scale, except at the night scale where generalized linear models were used. The AICc, ΔAICc, AICc weight (AICc.w) and sum of weights (sum.w) are given. Not all models tested are shown: only the best models are given.
(DOCX)
