# Supplementary material for: Can We Predict Foraging Success in a Marine Predator from Dive Patterns Only? Validation with Prey Capture Attempt Data
Source: PLoS One. 2014 Mar 6;9(3):e88503. doi: 10.1371/journal.pone.0088503 (PMC3945944; doi:10.1371/journal.pone.0088503)
Supplement: Table S1 — Results of model selection at every time scales. Models are generalized linear mixed models at all time scale, except at the night scale where generalized linear models were used. The AICc, ΔAICc, AICc weight (AICc.w) and sum of weights (sum.w) are given. Not all models tested are shown: only the best models are given. (DOCX) [file pone.0088503.s001.docx]

## Supporting Information

**Models details Table S1**: Results of model selection at every time scales. Models are generalized linear mixed models at all time scale, except at the night scale where generalized linear models were used. The AICc, ΔAICc, AICc weight (AICc.w) and sum of weights (sum.w) are given. Not all models tested are shown: only the best models are given.

**A-Dive scale**

|  |  |  |  |  |
| --- | --- | --- | --- | --- |
| Models | AICc | ΔAICc | AICc.w | sum.w |
| maximum dive depth + descent rate + ascent rate + PDI + DDS + AAS + BDS |  |  |  |  |
| … + Δ Depth at bottom + bottom duration | 6926,08 | 0 | 0,316 | 0,32 |
| … + Δ Depth at bottom + bottom duration + BW | 6927,52 | 1,43 | 0,155 | 0,47 |
| … + Δ Depth at bottom + bottom duration + BAS | 6927,61 | 1,52 | 0,148 | 0,62 |
| … + Δ Depth at bottom | 6928,15 | 2,07 | 0,112 | 0,73 |
| … + Δ Depth at bottom + BAS | 6928,90 | 2,81 | 0,078 | 0,81 |
| … + Δ Depth at bottom + bottom duration + BAS + BW | 6928,90 | 2,81 | 0,078 | 0,89 |
| … + Δ Depth at bottom + BW | 6929,28 | 3,19 | 0,064 | 0,95 |
| … + Δ Depth at bottom + BAS + BW | 6929,80 | 3,71 | 0,049 | 1,00 |
|  |  |  |  |  |
|  |  |  |  |  |

|  |  |  |  |  |
| --- | --- | --- | --- | --- |
| **B-Dive bout scale**   \| Models \| AICc \| ΔAICc \| AICc.w \| sum.w \| \| --- \| --- \| --- \| --- \| --- \| \| Nb dive + mean descent rate + mean ascent rate + mean BDS + mean BAS \|  \|  \|  \|  \| \| …+ mean bottom duration + mean AAS \| 442,02 \| 0,00 \| 0,941 \| 0,941 \| \| …+ mean Δ Depth at bottom + mean AAS \| 448,11 \| 6,09 \| 0,045 \| 0,986 \| \| …+ mean AAS \| 452,81 \| 10,79 \| 0,004 \| 0,990 \| \| …+ mean maximum dive depth + mean AAS \| 453,04 \| 11,02 \| 0,004 \| 0,994 \| \| …+ mean surface duration + mean AAS \| 455,17 \| 13,15 \| 0,001 \| 0,995 \| \| …+ % TD 15m + mean AAS \| 455,30 \| 13,28 \| 0,001 \| 0,997 \| \| …+ mean AAS + mean DDS \| 455,36 \| 13,34 \| 0,001 \| 0,998 \| \| …+ mean AAS + mean BW \| 455,38 \| 13,36 \| 0,001 \| 0,999 \| \| …+ mean maximum dive depth + mean bottom duration \| 456,77 \| 14,75 \| 0,001 \| 1,000 \| \|  \|  \|  \|  \|  \|   **C-30 min scale**   \|  \|  \|  \|  \|  \| \| --- \| --- \| --- \| --- \| --- \| \| Models \| AICc \| ΔAICc \| AICc.w \| sum.w \| \| Nb dive + % TD 15m + mean maximum dive depth + mean PDI + mean bottom duration + mean descent rate  + mean ascent rate + mean AAS \|  \|  \|  \|  \| \| ... + mean BDS + mean DDS + mean BAS \| 573,99 \| 0 \| 0,238 \| 0,24 \| \| ... + mean BDS + mean DDS \| 575,61 \| 1,63 \| 0,105 \| 0,34 \| \| ... + mean BDS + mean DDS + mean BAS + mean BW \| 575,92 \| 1,93 \| 0,09 \| 0,43 \| \| ... + mean BDS + mean Δ Depth at bottom + mean DDS + mean BAS \| 576,05 \| 2,07 \| 0,084 \| 0,52 \| \| ... + mean BDS + mean BAS \| 576,12 \| 2,13 \| 0,082 \| 0,60 \| \| ... + mean BDS \| 576,20 \| 2,21 \| 0,079 \| 0,68 \| \| ... + mean BDS + mean BDS + mean BW \| 577,17 \| 3,18 \| 0,048 \| 0,73 \| \| ... + mean BDS + mean BW \| 577,74 \| 3,76 \| 0,036 \| 0,76 \| \| ... + mean BDS + mean Δ Depth at bottom + mean DDS \| 577,83 \| 3,85 \| 0,035 \| 0,80 \| \| ... + mean BDS + mean BAS + mean BW \| 577,96 \| 3,97 \| 0,033 \| 0,83 \| \| ... + mean BDS + mean Δ Depth at bottom + mean DDS + mean BAS + mean BW \| 577,99 \| 4,01 \| 0,032 \| 0,86 \| \| ... + mean BDS + mean Δ Depth at bottom + mean BAS \| 578,27 \| 4,28 \| 0,028 \| 0,89 \| \| ... + mean BDS + mean Δ Depth at bottom \| 578,40 \| 4,41 \| 0,026 \| 0,92 \| \| ... + mean BDS + mean Δ Depth at bottom + mean DDS + mean BW \| 579,42 \| 5,43 \| 0,016 \| 0,93 \| \| ... + mean BDS + mean Δ Depth at bottom + mean BW \| 579,98 \| 5,99 \| 0,012 \| 0,94 \| \| ... + mean BDS + mean Δ Depth at bottom + mean BAS + mean BW \| 580,11 \| 6,13 \| 0,011 \| 0,96 \| \|  \|  \|  \|  \|  \| |  |  |  |  |

| **D-1 hour scale** |  |  |  |  |
| --- | --- | --- | --- | --- |
| Models | AICc | ΔAICc | AICc.w | sum.w |
| Nb dive + % TD 15m + mean maximum dive depth + mean PDI + mean bottom duration + mean descent rate + mean ascent rate |  |  |  |  |
| … + mean Δ Depth at bottom + mean AAS + mean BAS | 325,17 | 0 | 0,110 | 0,11 |
| … + mean Δ Depth at bottom + mean AAS + mean BW | 325,34 | 0,17 | 0,101 | 0,21 |
| … + mean Δ Depth at bottom + mean AAS | 325,39 | 0,22 | 0,098 | 0,31 |
| … + mean Δ Depth at bottom + mean AAS + mean BAS + mean BW | 326,28 | 1,11 | 0,063 | 0,37 |
| … + mean Δ Depth at bottom + mean AAS + mean DDS | 327,27 | 2,11 | 0,038 | 0,41 |
| … + mean Δ Depth at bottom + mean AAS + mean DDS + mean BW | 327,40 | 2,24 | 0,036 | 0,45 |
| … + mean Δ Depth at bottom + mean AAS + mean BDS + mean BAS | 327,42 | 2,25 | 0,036 | 0,48 |
| … + mean Δ Depth at bottom + mean AAS + mean DDS + mean BAS | 327,69 | 2,52 | 0,031 | 0,51 |
| … + mean Δ Depth at bottom + mean AAS + mean BDS + mean BW | 327,83 | 2,67 | 0,029 | 0,54 |
| … + mean AAS + mean BW | 327,84 | 2,67 | 0,029 | 0,57 |
| … + mean Δ Depth at bottom + mean AAS + mean BDS | 327,87 | 2,71 | 0,028 | 0,60 |
| … + mean AAS + mean BDS | 327,89 | 2,72 | 0,028 | 0,63 |
| … + mean AAS + mean BDS + mean BW | 328,30 | 3,13 | 0,023 | 0,65 |
| … + mean AAS | 328,62 | 3,45 | 0,020 | 0,67 |
|  |  |  |  |  |

| **E- 2 hours scale** |  |  |  |  |
| --- | --- | --- | --- | --- |
| Models | AICc | ΔAICc | AICc.w | sum.w |
| % TD 15m + mean bottom duration + mean descent rate + mean BDS | 119,53 | 0 | 0,452 | 0,45 |
| Nb dive + % TD 15m + mean bottom duration + mean descent rate | 119,69 | 0,16 | 0,417 | 0,87 |
| % TD 15m + mean PDI + mean bottom duration + mean descent rate | 123,20 | 3,67 | 0,072 | 0,94 |
| % TD 15m + mean maximum dive depth + mean bottom duration + mean descent rate | 126,13 | 6,61 | 0,017 | 0,96 |
| mean maximum dive depth + mean bottom duration + mean descent rate + mean BW | 127,66 | 8,13 | 0,008 | 0,97 |
| Nb dive + mean bottom duration + mean descent rate + mean BW | 127,72 | 8,19 | 0,008 | 0,97 |
|  |  |  |  |  |

| **F-Night scale** |  |  |  |  |
| --- | --- | --- | --- | --- |
| Models | AICc | ΔAICc | AICc.w | sum.w |
| mean maximum dive depth + mean ascent rate | 220,17 | 0 | 1 | 1 |
| mean maximum dive depth + mean AAS | 274,74 | 54,57 | 0 | 1 |
| % TD 15m + mean ascent rate | 288,10 | 67,93 | 0 | 1 |
| mean ascent rate + mean BDS | 290,79 | 70,61 | 0 | 1 |
| mean bottom duration + mean ascent rate | 295,27 | 75,10 | 0 | 1 |
| mean ascent rate + mean BW | 296,56 | 76,39 | 0 | 1 |
|  |  |  |  |  |
